# Intensive care unit presentation of central corneal descemetocele secondary to Methicillin-Resistant staphylococcal infection superimposed by a hypopyon: a case report

**DOI:** 10.1097/MS9.0000000000000327

**Published:** 2023-04-06

**Authors:** Vladislav P. Zhitny, Kyaw M. Tun, Katerina Roma, Gopi Narra, Jeremy P Kilburn

**Affiliations:** a Department of Internal Medicine; b Department of Critical Care Medicine, Kirk Kerkorian School of Medicine, Las Vegas, USA

**Keywords:** descemetocele, hypopyon, intensive care unit, methicillin-resistant staphylococcus

## Abstract

A descemetocele is a rare type of keratopathy that occurs when an intact descemet’s membrane of the eye undergoes a herniation through an overlying stroma. Previous literature has documented corneal damage via bacterial enzymes, especially, Pseudomonas and Neisseria species. Most recent prospective interventional studies showed treatment of these infections.

**Case presentation:**

This report presents the first instance of a methicillin-resistant *Staphylococcus aureus* descemetocele presentation in a 51-year-old African American male, with co-presenting hypopyon sequelae successfully managed conservatively in an intensive care unit setting.

**Clinical discussion:**

An instance of a methicillin-resistant *Staphylococcus aureus* has not yet been documented in the literature. Likewise, a co-presentation with a hypopyon, which is known as a formation of inflammatory debris rich in white blood cells has not been studied.

**Conclusion:**

The presence of a hypopyon in the instances of bacterial descemetocele herniation should be further evaluated to see if there are associations with conservative, nonsurgical intervention outcomes.

## Introduction

HIGHLIGHTS

- First reported methicillin resistance Methicillin-Resistant *Staphylococcus aureus*.
- Descemetocele co-presentation with a hypopyon may have a clinical correlate of clearing bacterial infection.
- *Staphylococcus aureus* descemetocele presentation co-presenting hypopyon successful conservative management in an intensive care unit setting.

Keratopathy was reported to occur in 6–57% of ICU patients around the world^1^,^2^. A particularly rare complication is that of a descemetocele, which occurs when an intact descemet membrane undergoes anterior herniation through an overlying stroma. This is an ophthalmologic emergency due to the risk of perforation^3^. Due to the rarity of this condition, there is limited literature available. Etiologies range from trauma, iatrogenic, immune-related, neurotrophic keratitis, and microbial keratitis^3^. While there is no established consensus for the management of descemetocele, much of the current literature recommends surgical intervention rather than a conservative management^3^–^6^. Bacterial cases reported in the literature have been that of *Pseudomonas* and *Neisseria* species^3^,^7^,^8^.

A hypopyon is an inflammatory condition of the eye that involves a sediment of white blood cells of the anterior chamber. The classic white exudate-rich fluid, usually accompanied by associated conjunctival redness, is constituted from white blood cells secondary to inflammation of the iris and uvea^9^. The current practice is not to drain an accumulated hypopyon due to risks of synechiae and closed-angle glaucoma^10^,^11^.

We present the case of a descemetocele caused by Methicillin-Resistant *Staphylococcus aureus* (MRSA) and a co-existent hypopon, neither of which have been previously described. Of note, the work has been reported in line with the CARE criteria^12^. A signed written informed consent was obtained from the patient pertaining to the release of protected health information and photographs prior to commission of this case report.

## Case presentation

**Figure 1:**
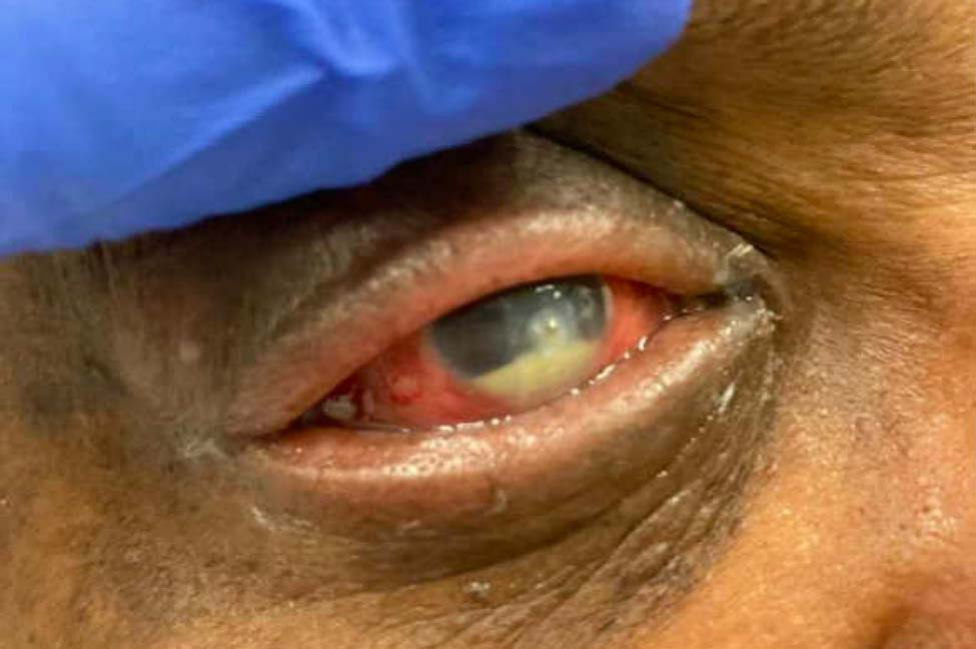
Descemetocele superimposed by a hypopyon (day 1).

**Figure 2:**
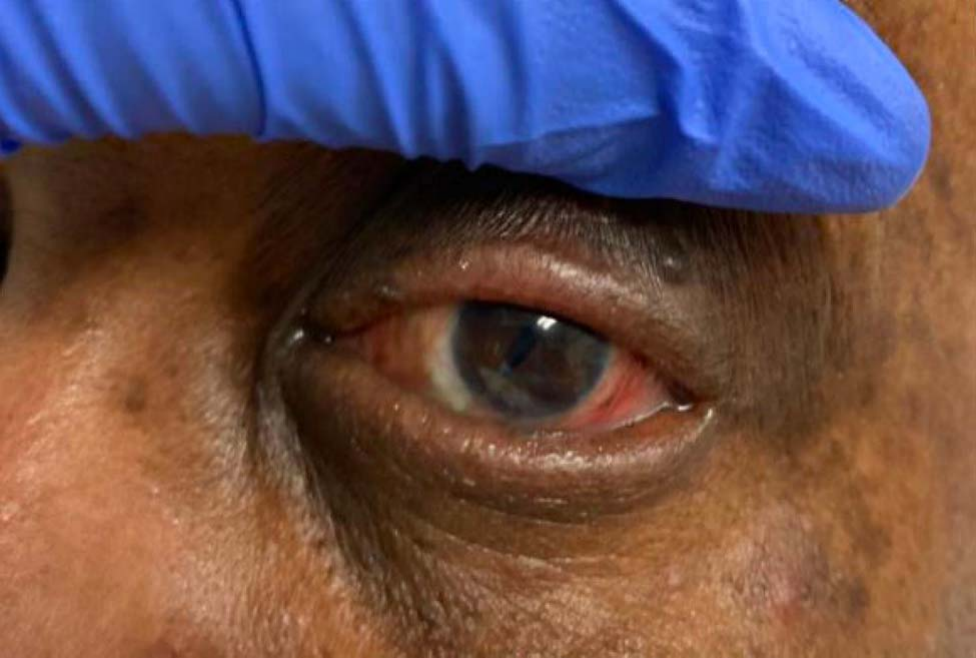
Unaffected left eye without presence of descemetocele.

**Figure 3:**
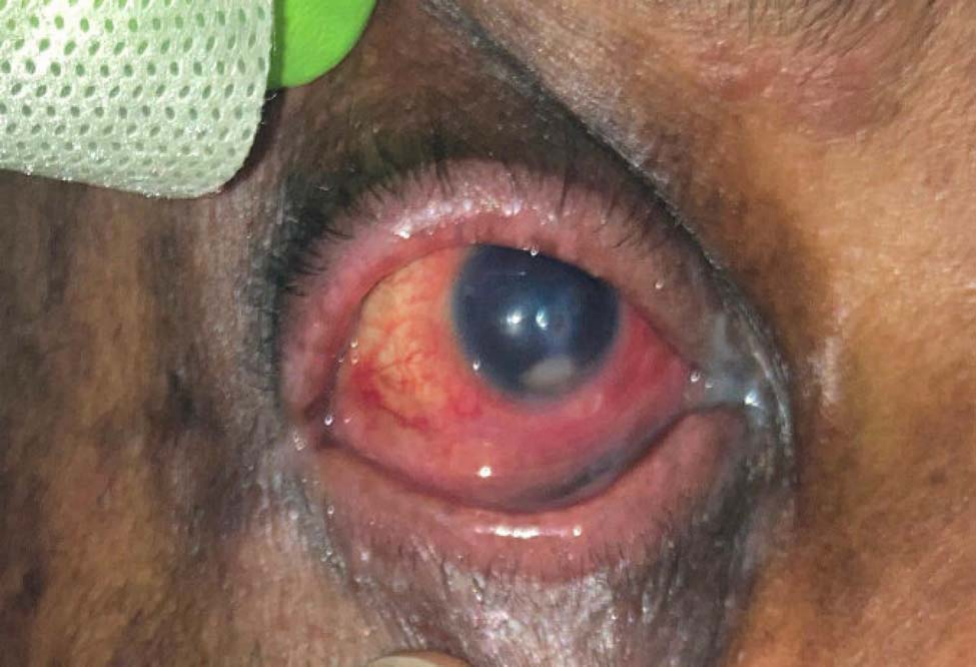
Descemetocele superimposed by a hypopyon on (day 13).

A 51-year-old African American male with a past medical history of glaucoma in the left eye and type 2 diabetes mellitus, presented with worsening right eye pain for 2 weeks. He was previously seen at an outpatient clinic for ocular pruritus on the right eye and treated with fortified cefazolin and tobramycin ophthalmic solutions. However, the patient’s symptoms worsened, prompting his presentation to the emergency department. On physical exam, the patient’s pupils were round and equally reactive to light. The intraocular pressure was measured to be 12 and 10 mmHg in the right and left eyes, respectively. Slit lamp examination revealed 3+ conjunctival injection on the right (Fig. 1). The right cornea demonstrated a 2 mm central descemetocele surrounded by 6 mm central corneal infiltrate. Corneal folds were present in the right eye. Additionally, hypopyon was noted in the anterior chamber and occupied 30% of the right eye. The iris of the right eye was round and regular without neovascularization. Pseudophakia was present in both eyes. A dilated fundus exam was difficult to obtain due to corneal ulcer and corneal folds and was not performed. There was no evidence of viritis. Examination of the left eye showed scleral injection but was otherwise unremarkable (Fig. 2). The patient was diagnosed with a central corneal descemetocele on the right eye and was subsequently admitted to the ICU for hourly eye exams. The laboratories at the time of the admission demonstrated an elevated white blood cell count 10.64 (3.10–10.20 k/mm^3^). The eye culture swab showed growth of MRSA resistant to penicillin, oxacillin, clindamycin, and linezolid. The patient received hourly antibiotic administration with fortified cefazolin, tobramycin, and fortified vancomycin ophthalmic drops. Furthermore, he was treated with systemic intravenous vancomycin for potential systemic source control and treatment of hypopyon for a total of 9 days. After 13 days of treatment, localized swelling around the right eye improved and the leukocytosis resolved (Fig. 3). His need for ophthalmic solutions decreased from every hour to every 3 h. The patient was subsequently downgraded from the ICU.

## Discussion

The pathologic process of bacterial descemetocele involves damage to the cornea secondary to the proteolytic activity of bacterial enzymes and other toxins^13^,^14^. The first study that elucidated the causes and management of descemetoceles was published in 1984 in the journal of *Transactions of the American Ophthalmological Society* 15. The most recent prospective interventional study published in 2022 by Shankar *et al*.^16^ included a total of 24 patients and discussed the importance of fortified antibiotic administration and the possible need for surgical intervention. In case should conservative therapy fail, a surgical intervention may be required in which an amniotic membrane transplant and blepharorrhaphy are performed. Bacterial keratitis descemetocele have been described with *Pseudomonas* and *Neisseria* species infections^7^,^8^. In one case report regarding descemetocele secondary to *Neisseria* species, the patient failed medical management with an ophthalmic antibiotic therapy and required surgical intervention via a deep anterior lamellar keratoplasty. Despite the intervention, the patient’s condition worsened and required lamellar anterior keratoplasty.

Our experience of a descemetocele secondary to MRSA infection with hypopyon followed a similar clinical course as those of other microbial descemetocele presentations previously described. However, this case differs from previous reports by a newly reported infection from MRSA, not requiring surgical intervention, and a presence of a hypopyon. By definition, hypopyon is a formation of inflammatory debris rich in white blood cells and may have a clinical correlate of clearing bacterial infection in the instances of a descemetocele. It prevents progression of anterior descemet membrane herniation through an overlying stroma. Although the risks of a hypopyon-related complications (e.g. acute angle glaucoma due to limited space in the anterior chamber of the eye) should be weighed, the presence of a hypopyon in the instances of bacterial descemate herniation should be further evaluated to see if there are associated better outcomes. This work has been reported in line with the Surgical CAse REport (SCARE) 2020 Criteria^12^.

## Ethical approval

Ethical approval was permitted by the hospital Institutional Review Board.

## Consent

Authors received permission from the patient pertaining release of protected health information, photograph, and video release prior to writing of the case study.

## Sources of funding

No external or internal funding influenced the conduction of this study.

## Author contribution

V.P.Z. was involved in concept design, writing the paper, and literature assessment; K.M.T. assisted in writing case presentation; G.N. assisted in writing introduction; K.R. assisted in writing discussion; J.K. is the principal investigator and the guarantor physician for this study.

## Conflict of interest disclosure

Authors have no conflicts of interests to disclose with this case report.

## Research registration unique identifying number (UIN)

Does not apply.

## Guarantor

Jeremy Kilburn, MD; University Medical Center of Southern Nevada.

## Provenance and peer review

Not commissioned, externally peer reviewed.

## References

[1] Kuruvilla S Peter J David S . Incidence and risk factor evaluation of exposure keratopathy in critically ill patients: a cohort study. J Crit Care 2015;30:400–404. 25468364 10.1016/j.jcrc.2014.10.009

[2] Hsieh YC Chen CC . Descemetocele and bilateral, severe Pseudomonas keratitis in an intensive care unit patient with Graves’ orbitopathy: a case report. Medicine (Baltimore) 2020;99:e22481. 33019441 10.1097/MD.0000000000022481 PMC7535751

[3] Agarwal R Nagpal R Todi V . Descemetocele. Surv Ophthalmol 2021;66:2–19. 33058926 10.1016/j.survophthal.2020.10.004

[4] Ozdemir ES Burcu A Akkaya ZY . Surgical outcomes of perforated and unperforated corneal descemetocele. Int Ophthalmol 2018;38:327–335. 28224301 10.1007/s10792-017-0472-z

[5] Sharma N Kumar C Mannan R . Surgical technique of deep anterior lamellar keratoplasty in descemetoceles. Cornea 2010;29:1448–1451. 20847654 10.1097/ICO.0b013e3181e2ef9c

[6] Portnoy SL Insler MS Kaufman HE . Surgical management of corneal ulceration and perforation. Surv Ophthalmol 1989;34:47–58. 2678553 10.1016/0039-6257(89)90129-x

[7] Mohammadpour M Sabet FA . Long-term outcomes of amniotic membrane transplantation in contact lens-induced pseudomonas keratitis with impending corneal perforation. J Ophthalmic Vis Res 2016;11:37–41. 27195083 10.4103/2008-322X.180712 PMC4860985

[8] Tong L Tan DT Abańo JM . Deep anterior lamellar keratoplasty in a patient with descemetocele following gonococcal keratitis. Am J Ophthalmol 2004;138:506–507. 15364250 10.1016/j.ajo.2004.04.014

[9] Gaudio PA, & Huang JJ (2010). *Ocular inflammatory disease and uveitis manual: Diagnosis and treatment*. Wolters Kluwer-Lippincott Williams & Wilkins.

[10] Sprabary A . (2022, June 15). *Narrow-angle glaucoma (angle-closure glaucoma)*. All About Vision. Accessed 8 July 2022. https://www.allaboutvision.com/conditions/narrow-angle-glaucoma/

[11] Maria Hannah Pia U de Guzman MD . (2021, July 19). *Peripheral Anterior Synechia Treatment & management: Approach considerations, medical care, Surgical Care*. Peripheral Anterior Synechia Treatment & Management: Approach Considerations, Medical Care, Surgical Care. Accessed 8 July 2022. https://emedicine.medscape.com/article/1189962-treatment

[12] Agha RA Franchi T Sohrab C . The CARE 2020 guideline: updating consensus Surgical Case Report (CARE) guidelines. Int J Surg 2020;84:226–230. 33181358 10.1016/j.ijsu.2020.10.034

[13] Sheha H Liang L Li J . Sutureless amniotic membrane transplantation for severe bacterial keratitis. Cornea 2009;28:1118–1123. 19770726 10.1097/ICO.0b013e3181a2abad PMC2846111

[14] Chen JH Ma DH Tsai RJ . Amniotic membrane transplantation for pseudomonal keratitis with impending perforation. Chang Gung Med J 2002;25:144–152. 12022734

[15] Arentsen JJ Laibson PR Cohen EJ . Management of corneal descemetoceles and perforations. Trans Am Ophthalmol Soc 1984;82:92–105. 6398940 PMC1298656

[16] Shankar S Agarwal R Nagpal R . Management of descemetocele: our experience and a simplified treatment algorithm. Indian J Ophthalmol 2022;70:1564–1570. 35502027 10.4103/ijo.IJO_3070_21 PMC9332962

